# Supplementary material for: Orbital-dependent electron correlation in double-layer nickelate La3Ni2O7
Source: Nat Commun. 2024 May 23;15:4373. doi: 10.1038/s41467-024-48701-7 (PMC11116484; doi:10.1038/s41467-024-48701-7)
Supplement: Supplementary file 1 — Supplementary Information [file 41467_2024_48701_MOESM1_ESM.pdf]

Supplementary Information:

Orbital-Dependent Electron Correlation in Double-Layer Nickelate $La_3Ni_2O_7$

Jiangang Yang[1,2,♯], Hualei Sun[3,♯], Xunwu Hu[4,♯], Yuyang Xie[1,2], Taimin Miao[1,2], Hailan Luo[1,2], Hao Chen[1,2], Bo Liang[1,2], Wenpei Zhu[1,2], Gexing Qu[1,2], Cui-Qun Chen[4], Mengwu Huo[4], Yaobo Huang[5], Shenjin Zhang[6], Fengfeng Zhang[6], Feng Yang[6], Zhimin Wang[6], Qinjun Peng[6], Hanqing Mao[1,2,7], Guodong Liu[1,2,7], Zuyan Xu[6], Tian Qian[1,2,7], Dao-Xin Yao[4,*], Meng Wang[4,*], Lin Zhao[1,2,7,*] and X. J. Zhou[1,2,7,*]

[1] *Beijing National Laboratory for Condensed Matter Physics, Institute of Physics, Chinese Academy of Sciences, Beijing 100190, China.*

[2] *School of Physical Sciences, University of Chinese Academy of Sciences, Beijing 100049, China.*

[3] *School of Science, Sun Yat-Sen University, Shenzhen, Guangdong 518107, China.*

[4] *Guangdong Provincial Key Laboratory of Magnetoelectric Physics and Devices, School of Physics, Sun Yat-Sen University, Guangzhou 510275, China.*

[5] *Shanghai Synchrotron Radiation Facility, Shanghai Advanced Research Institute, Chinese Academy of Sciences, Shanghai 201204, China.*

[6] *Technical Institute of Physics and Chemistry, Chinese Academy of Sciences, Beijing 100190, China.*

[7] *Songshan Lake Materials Laboratory, Dongguan, Guangdong 523808, China.*

[♯] *These people contribute equally to the present work.*

[*] *Corresponding authors: XJZhou@iphy.ac.cn, LZhao@iphy.ac.cn, wangmeng5@mail.sysu.edu.cn, yaodaox@mail.sysu.edu.cn*

Supplementary Note 1. Orbital characters of band structures in $La_3Ni_2O_7$ based on polarization-dependent ARPES measurements

To investigate the orbital characters of different bands in $La_3Ni_2O_7$, we carried out polarization-dependent ARPES measurements as summarized in Supplementary Fig. 1. Supplementary Fig. 1a-c and Supplementary Fig. 1d-f show the results measured under the *s*- and *p*-polarization geometries, respectively. In the *s*-polarization geometry, the electric field vector E of the incident light is perpendicular to the photoelectron emission plane; its direction is marked by the double arrow near the bottom-left corner. In the *p*-polarization geometry, the electric field vector E is within the photoelectron emission plane. In our case, it consists of both the in-plane component and the out-of-plane component.

The spectral intensity in ARPES measurements depends on the photoemission matrix element effects[1]. The matrix element M is determined by $<\phi_f^k|\mathbf{A}\cdot\mathbf{p}|\phi_i^k>$, where$|\phi_i^k>$ is the initial state, $|\phi_f^k>$ represents the final state, $\mathbf{A}$ is the electromagnetic vector potential and $\mathbf{p}$ is the electron momentum operator. Since the final state is treated as being even, the matrix then depends on the parity product of the light polarization and the initial state. The parity of the light polarization and the initial state can be determined with respect to a given mirror plane.

In our polarization-dependent measurements (Supplementary Fig. 1), we define the mirror plane along the $\bar{\Gamma}$-$\bar{X}$ direction (red line in Supplementary Fig. 1a) and perpendicular to the sample surface. The $d_{x^2-y^2}$ is odd and d_{z^2} is even with respect to the defined mirror plane. The E vector is odd in the *s*-polarization geometry while it is even in the *p*-polarization geometry. Therefore, in the *s*-polarization geometry, $d_{x^2-y^2}$ is allowed while d_{z^2} is forbidden. On the other hand, in the *p*-polarization geometry, $d_{x^2-y^2}$ is forbidden while d_{z^2} is allowed. These analyses are summarized in Supplementary Table I.

In the *s*-polarization geometry, along the $\bar{\Gamma}$-$\bar{X}$ direction, the α/β Fermi surfaces (Supplementary Fig. 1a) and band structures (Supplementary Fig. 1c) are clear and strong. In the *p*-polarization geometry, the α/β Fermi surfaces (Supplementary Fig. 1d) and band structures (Supplementary Fig. 1f) get strongly surpressed. On the other hand, in the *s*-polarization geometry, near (π,π), the γ feature (Supplementary Fig. 1b) and γ band (Supplementary Fig. 1c) are hardly visible. In the *p*-polarization geometry, the γ feature (Supplementary Fig. 1e) and γ band (Supplementary Fig. 1f) become clearly observable. Based on the above matrix element analysis, these results are consistent with the orbital

assignment that the α/β bands are dominated by the Ni-3d$_{x^2-y^2}$ orbital while the γ band is dominated by the Ni-3d$_{z^2}$ orbital.

Supplementary Table I: **Parity of the E-vector and orbitals with respect to the $\bar{\Gamma}$-$\bar{X}$ mirror plane**

	***s*-pol.** E-vector:odd	***p*-pol.** E-vector:even
d$_{x^2-y^2}$	odd, allowed	odd, forbidden
d$_{z^2}$	even, forbidden	even, allowed

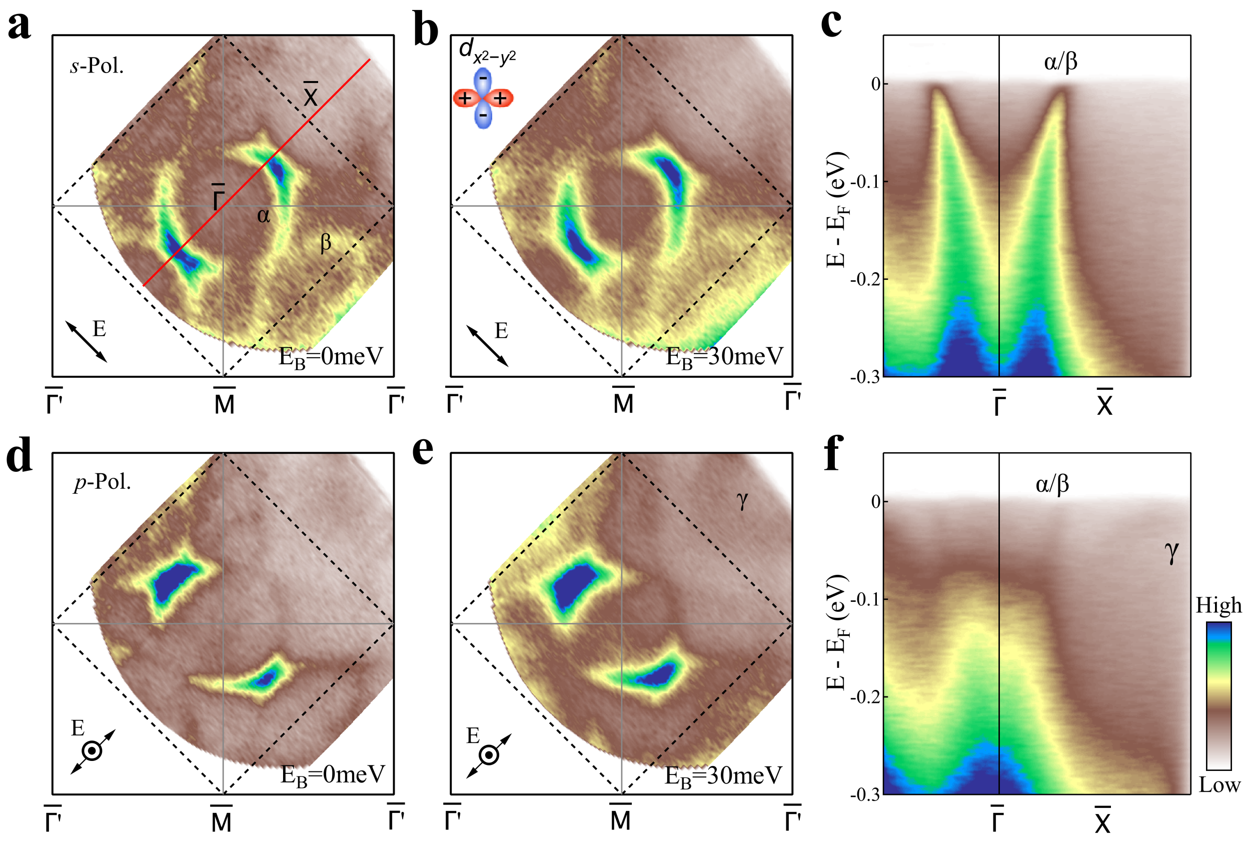

Supplementary Figure 1: Polarization-dependent ARPES measurements of $La_3Ni_2O_7$. **a** Fermi surface mapping measured under the s polarization geometry. The direction of the corresponding electric field vector E is marked by a double arrow near the bottom-left corner. **b** The corresponding constant energy contour at the binding energy of 30 meV. **c** The corresponding band structure measured along the $\bar{\Gamma}$-$\bar{X}$ direction. The location of the momentum cut is shown by the red line in **a**. **d-f** Same as **a-c** but measured under the p polarization geometry. The direction of the corresponding electric field vector E consists of both in-plane component and out-of-plane component. In the upper-left inset of **b**, the schematic $d_{x^2-y^2}$ orbital is shown.

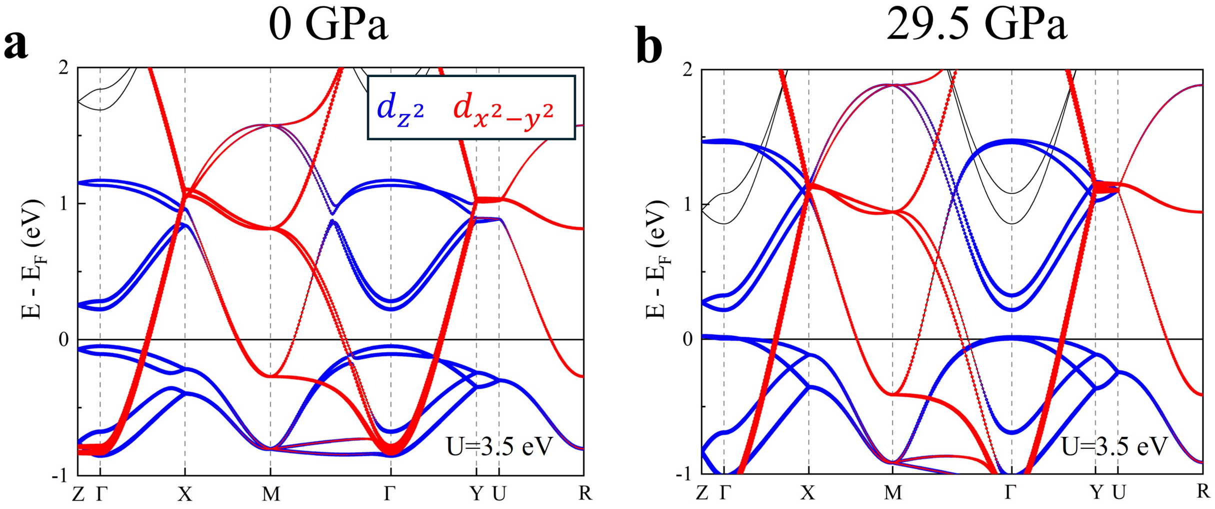

Supplementary Figure 2: Calculated band structures of $La_3Ni_2O_7$ with U=3.5 eV at 0 GPa (a) and 29.5 GPa (b).

Supplementary References

---

[1] Damascelli, A., Hussain, Z. and Shen, Z.-X. Angle-resolved photoemission studies of the cuprate superconductors. *Rev. Mod. Phys* **75**, 473 (2003).
